# Bone mineral density and microarchitecture linkages with micro- and macro-vascular impairments at the hand in systemic sclerosis: an HRpQCT study

**DOI:** 10.18632/oncotarget.25681

**Published:** 2018-06-29

**Authors:** Lucie Atlan, Nada Ibrahim-Nasser, Antoine Valery, Carole Bazzi, François Rollin, Guido Bens, Mathilde Marot, Eric Estève, Eric Lespessailles

**Affiliations:** ^1^ Department of Rheumatology, University Hospital of Tours, Tours, France; ^2^ Department of Rheumatology, Regional Hospital of Orleans, Orleans, France; ^3^ University of Orleans, I3MTO Laboratory, Orleans, France; ^4^ Department of Medical Information, Regional Hospital of Orleans, Orleans, France; ^5^ Department of Vascular Medicine, Regional Hospital of Orleans, Orleans, France; ^6^ Department of Dermatology, Regional Hospital of Orleans, Orleans, France

**Keywords:** systemic sclerosis, high-resolution peripheral quantitative computed tomography, bone microarchitecture, vasculopathy, nailfold videocapillaroscopy

## Abstract

**Objective:**

To investigate the link between bone alteration and micro- and macro-vascular disease in patients with systemic sclerosis (SSc).

**Results:**

33 SSc patients were included. In univariate analysis, low values of cortical vBMD were significantly associated with a low DBI at the 2nd finger (*p* = 0.004) and at the 4th (*p* = 0.002) and with severe capillaroscopic score (*p* = 0.008). In multivariate analyses, low cortical vBMD was associated with a low DBI at the 4th finger, age and severe capillaroscopic score (adjusted R^2^ = 0.58; *p* = < 0.001). Low cortical thickness was associated with a low DBI at the 4th finger, severe capillaroscopic score and age (adjusted R^2^ = 0.49, *p* = < 0.001).

**Conclusion:**

Our study findings showed an association between micro- and macro-vessel damage and altered microarchitectural indices at the radius in SSc.

**Methods:**

We performed a pilot study in female patients with SSc. Microvascular disease was assessed by the capillaroscopic score of Cutolo. Macrovascular involvement was measured by digito-brachial pressure index (DBI) on laser-Doppler at the 2nd and 4th finger. Volumetric bone mineral density (vBMD) and bone microarchitecture involvement were analysed by High-Resolution peripheral Quantitative Computed Tomography (HRpQCT) at the distal radius.

## INTRODUCTION

Systemic sclerosis (SSc) is a connective tissue disease of unknown etiology characterized by immunological disturbances, microangiopathy, and fibrosis of tissues and vessels, which can cause failure of vital organs [1]. The vascular abnormalities are defined by a dysfunction of the endothelium [2], with an inability to control the inflammation leading to a recruitment of inflammatory cells in the perivascular areas and to a permanent connective tissue remodeling, causing fibrosis [2]. This results in an obliteration of the vascular lumen inducing ischemia [3, 4].

The vascular involvement is the main prognostic factor of the SSc, because extension and severity of vascular lesions influence the disease progression [5]. Raynaud's phenomenon, frequently the first symptom of SSc [1], is a sign of microvascular involvement, as well as digital ulcers. Peripheral vascular involvement with alteration of medium caliber arteries of the hand has been known for a long time [6]. In an angiographic study, occlusion of the ulnar arteries was found in half of the patients suffering from systemic sclerosis, responsible for digital trophic disorders and Raynaud's phenomenon [6]. The non-invasive detection of these lesions is now possible thanks to Doppler flowmeter and ultrasound with high frequency probe or angio-MRI [7].

In addition to skin and vascular involvement, musculoskeletal manifestations of SSc are also recognized to be associated with significant disability [8, 9]. More recently, increased risks of osteoporosis and fractures associated with SSc have been underlined [10–14]. We have reported that digital ulcers were independent risk factors for bone damage, using for the first time a High Resolution peripheral Quantitative Computed Tomography device (HRpQCT) to investigate the bone status [15]. HRpQCT is a new and most promising 3D imaging technique which has been primarily developed to explore the quality of bone [16]. However thanks to its capacity to image fine details (75 to 100 μm) emerging data on Rheumatoid Arthritis and connective tissue disease also exists [16].

Beyond the specific associations that can be found between osteoporosis and cardiovascular disease in systemic inflammatory autoimmune disease [17], there is a great body of evidence in epidemiologic studies suggesting an association between osteoporosis and cardiovascular diseases [18]. More specifically, a selective bone loss at the radius has recently been observed in patients with subclinical peripheral arterial disease (PAD) as compared to controls [19].

Therefore, in the present study, we investigated the association between volumetric Bone Mineral Density (vBMD) using a HRpQCT device and both microvascular and macrovascular alterations at the upper limbs in a group of women with SSc.

## RESULTS

### Descriptive analysis

33 SSc patients were included in the study. The characteristics of subjects included in the study are described in Table 1. In Table 2 are presented the results of nailfold videocapillaroscopy, vascular evaluation by Medsger's score, and laser-Doppler ultrasonography. Patients were all right-handed, that is why the DBI at the left hand (non-dominant hand) were selected for the vascular evaluation. The results of bone densitometric and microarchitectural variables by HRpQCT at the radius in SSc patients are described in Table 3. One patient was excluded because of motion artefacts on HRpQCT.

**Table 1:** Characteristics of subjects included in the study

**Demographics**	
** Age, years (mean ± SD)**	63.2 ± 10.2
** BMI, kg/m^2^ (mean ± SD)**	24.7 ± 4.9
** Duration of menopause, years (mean ± SD)**	15.4 ± 11.4
** Menopause, *n* (%)**	28 (85)
** Current smoking, *n* (%)**	2 (6)
** Alcohol, *n* (%)**	0 (0)
** High blood pressure, *n* (%)**	10 (30.3)
** Diabetes, *n* (%)**	2 (6.1)
** Dyslipidemia (*n* = 32), *n* (%)**	6 (18.8)
** Personal history of fracture, *n* (%)**	10 (30.3)
** Family history of hip fracture, *n* (%)**	5 (15.1)
**Treatment, *n* (%)**	
** Prednisone**	6 (18.2)
** Osteoporosis treatment**	12 (36.4)
** Calcium and/or vitamin D**	20 (60.6)
** Inhibitor of proton pump**	26 (78.8)
** Methotrexate**	2 (6)
** Hydroxychloroquine**	7 (21.2)
** History of cyclic intravenous prostanoids**	10 (30.3)
** Cyclophosphamide**	0 (0)
**Biology, *n* (%)**	
** CRP (>10 mg/l)**	1 (3)
** Vitamin D deficiency (<10 ng/ml)**	2 (6)
** Vitamin D insufficiency (10–30 ng/ml)**	18 (54.5)
** Parathyroid hormone (>61 ng/l)**	4 (12.1)
** Calcemia (<2.2 or >2.6 mmol/l)**	0 (0)
** ANA**	33 (100)
** ACA**	20 (60.6)
** Antitopoisomerase (anti-Scl70)**	4 (12.1)
** Anti-RNA polymerase III**	0 (0)
**Disease Characteristics**	
** Disease duration, years (mean ± SD)**	9.5 ± 8.4
** Rodnan modified score (*n* = 31), mean ± SD**	6.39 ± 6.66
** Skin involvment subset, *n* (%)**	
** limited cutaneous**	26 (78.8)
** diffuse cutaneous**	3 (9.1)
** limited sine scleroderma**	4 (12.1)
** Current digital ulcers, *n* (%)**	12 (36.4)
** History of digital ulcers, *n* (%)**	21 (63.6)
** Organ involvment, *n* (%)**	
** gastrointestinal involvment**	24 (72.7)
** malabsorption syndrome**	0 (0)
** lung disease**	9 (27.2)
** pulmonary hypertension**	4 (12.1)
** scleroderma renal crisis**	0 (0)
** joint damage**	26 (78.8)
** Raynaud's syndrome**	33 (100)
** HAQ (*n* = 32) (mean ± SD)**	0.833 ± 0.830

Abbreviations: ACA: anticentromere antibody; ANA: anti-nuclear antibody; BMI: body mass index; CRP: C reactive Protein; HAQ: Health Assessment Questionnaire; n: number; SD: standard deviation.

**Table 2:** Results of Nailfold videocapillaroscopy, Evaluation by Medsger's score and laser-Doppler ultrasound of the upper limbs

Variables, *n* (%)	SSc patients (*n* = 33)
**Clinical evaluation by Medsger's score**	
Non severe	16 (48.5)
no Raynaud syndrome or Raynaud syndrome not requiring vasodilators (0)	13 (39.4)
Raynaud syndrome requiring vasodilatators (1)	3 (9.1)
Severe	17 (51.5)
digital pitting scars (2)	3 (9.1)
digital tip ulcerations (3)	13 (3.4)
digital gangrene (4)	1 (3.0)
**Nailfold videocapillaroscopic evaluation by Cutolo's score**	
Non severe	20 (60.6)
0 : normal pattern	0 (0%)
I : early pattern	20 (60.6)
Severe	13 (39.4)
II : active pattern	9 (27.3)
III : late pattern	4 (12.1)
**Evaluation by laser-Doppler ultrasound of the upper limbs**	
DBI ≤ 0.7	
Left second finger	4 (12.1)
Left fourth finger	8 (24.2)
Radial artery involvement (stenosis or occlusion)	0 (0)
unilateral	0 (0)
bilateral	0 (0)
Ulnar artery involvement (stenosis or occlusion)	4 (12.1)
unilateral	3 (9.1)
bilateral	1 (3.0)
Palmar arches involvment (stenosis or occlusion)	9 (27.3)
unilateral	5 (15.2)
bilateral	4 (12.1)

Abbreviations: DBI: digito-brachial pressure index; n: number.

**Table 3:** Results [mean (SD)] of bone densitometric and microarchitectural variables by HRpQCT at the radius in SSc patients

HRpQCT variables	SSc patients (*n* = 32)
**Tb.BMD, mgHA/cm^3^**	126 (42)
**Tt.BMD, mgHA/cm^3^**	288 (76)
**Ct.BMD, mgHA/cm^3^**	836 (87)
**BV/TV, %**	0.12 (0.11)
**Tb.N, mmˉ¹**	1, 56 (0.38)
**Tb.Th, μm**	67 (11)
**Tb.Sp, μm**	625 (232)
**TbSp.SD, μm**	324 (231)
**Ct.Th, μm**	712 (221)

Abbreviations: HRpQCT: High-Resolution peripheral Quantitative Computed Tomography; BV/TV: trabecular bone volume; Ct.BMD: cortical volumetric bone mineral density; Ct.Th: cortical thickness; n: number; Tb.BMD: trabecular volumetric bone mineral density; Tb.N: trabecular number; Tb.Sp: trabecular bone separation; Tb.Sp.SD: intra-individual distribution of separation; Tb.Th: trabecular thickness; Tt.BMD: total volumetric bone mineral density.

### Univariate analysis

Cortical vBMD (Ct.BMD) at the radius was associated with a Cutolo score ≥ stage II (*p* = 0.008) and with a DBI ≤ 0.7 at the second and fourth fingers (*p* = 0.035 and *p* = 0.002 respectively). Cortical thickness (Ct.th) at the radius was associated with the Cutolo's score and with the DBI at the fourth finger (*p* = 0.009 and *p* = 0.021 respectively). Trabecular thickness (Tb.th) at the radius was associated with the Medsger score (*p* = 0.008), as well as the DBI of the fourth fingers (*p* = 0.003 respectively). The association between vascular parameters and HRpQCT derived bone parameters is shown in Table 4.

**Table 4:** Univariate analysis

Variables	Cutolo's score ≥ pattern II, *p* value	Medsger's score ≥ 2, *p* value	DBI at second finger ≤ 0.7, *p* value	DBI at fourth finger ≤ 0.7, *p* value
**Tt.BMD**	0.077	**0.019**	0.123	**0.048**
**Ct.BMD**	**0.008**	0.089	0.035^*^	0.002^*^
**Tb.BMD**	0.631	**0.027**	0.36	**0.029**
**Tb.N**	0.553	0.239	0.878	0.330
**Tb.Sp**	0.909	0.216	1	0.366
**TbSp.SD**	0.514	0.146	0.35	**0.044**
**BV/TV**	0.564	**0.032**	0.276	**0.031**
**Ct.Th**	**0.009**	0.083	0.118	**0.021**
**Tb.Th**	0.901	**0.008**	0.177	0.003^*^

Relationship between bone parameters measured by HRpQCT at the radius and vascular parameters.

To consider multiple comparisons, the significance level must be adjusted with Holm correction for each vascular parameters.

Significance for univariate relations is flagged with^*^.

Factors selection into linear models is indicated with bold typing.

The results are given with the *p* value.

Abbreviations: BV/TV: trabecular bone volume; Ct.BMD: cortical volumetric bone mineral density; Ct.Th: cortical thickness; DBI: Digito-brachial index; n: number; Tb.BMD: trabecular volumetric bone mineral density; Tb.N: trabecular number; Tb.Sp: trabecular bone separation; Tb.Sp.SD: intra-individual distribution of separation; Tb.Th: trabecular thickness; Tt.BMD: total volumetric bone mineral density.

There was no significant association between cardiovascular risk (high blood pressure, diabetes, current smoking and dyslipidemia) and vascular parameters assessed in our study (DBI, capillaroscopic score of Cutolo and Medsger's score).

### Multivariate analysis

To test further our hypothesis that cortical bone parameters at the radius might be related to vascular parameters, we performed a multivariate analysis. The analysis showed that the variables influencing independently a decrease of cortical Ct.BMD at the radius were a DBI ≤ 0.7 at the 4th finger (*p* = 0.04), age (*p ≤* 0.001) and severe capillaroscopic score (*p* = 0.37) (adjusted R^2^ = 0.58; *p ≤* 0.001). The variables independently associated with a decrease of Ct.Th at radius were a DBI ≤ 0.7 at the 4th finger (*p* = 0.21), severe capillaroscopic score (*p* = 0.09) and age (*p* = 0.0006) (adjusted R^2^ = 0.49; *p* < 0.001). The variable associated with the decrease of Tb.Th at the radius was a DBI ≤ 0.7 at the 4th finger (*p* = 0.001) (R^2^ adjusted = 0.29; *p ≤* 0.001).

## DISCUSSION

The association between low BMD and atherosclerosis has been demonstrated in both epidemiological and prospective studies [20–23]. The inverse relation between vascular damages and BMD parameters might be attributable to shared common etiology such as tobacco use, vitamin D, age, diabetes or hypertension [24]. However, the novel finding in our cross sectional study of SSc patients is the observation that we found a significant and independent relationship between vascular involvement, as measured by the DBI at the fourth finger and the capillaroscopic score of Cutolo, with alterations of bone tissue measured by HRpQCT at the radius.

PAD has been identified as the earliest form of macrovascular disease in scleroderma [25]. Ulnar artery involvement (stenosis or occlusion) is the preferential site of vascular damage at the upper limb in SSc patients and the present study extends several reports of the literature showing that ulnar artery was affected at higher rate than other arteries in SSc [26–28]. Macrovascular disease, however, has also been reported in other rheumatic diseases with various angiogenesis and vasculogenesis processes associated with defective vascular repair [2, 29]. Evidence exits to support the link between subclinical atherosclerosis and osteoporosis [22, 23], and more specifically between PAD and osteoporosis or low BMD [18, 30–33]. Higher rates of hip BMD loss and an increased risk of non-vertebral fracture were reported in a prospective study including community dwelling older men with PAD [32]. In the Study of Osteoporotic Fracture, an increased rate of bone loss at the calcaneus and at the total hip was observed in healthy older women with decreased vascular flow in the lower extremities [18].

In most of the studies providing a link between the cardiovascular system and loss of bone and osteoporosis, the assessment of BMD was done by conventional dual energy X-ray absorptiometry devices that permitted areal BMD but not volumetric BMD measurements [17, 20]. In addition, computed tomography and HRpQCT devices now allow separate analysis of the trabecular and cortical compartment [16].

Relationship between atherosclerosis and osteoporosis has been investigated in post-menopausal women using Computed Tomography both in a North American population [21] and in an Italian one [31]. However, the results of these studies were not consistent, one showing no relationship between volumetric vertebral BMD nor between surfacic total hip BMD and PAD [21], whereas in the Italian study, PAD indices and bone cross-sectional area at the tibia were associated [31].

More recently, a selective bone loss assessed by pQCT in the cortical compartment was observed at the radius in patients with ankle brachial index <0.9 as compared to controls with ankle brachial index >0.9 [19]. In this last study, including both a small group of men (*n* = 14) and women (*n* = 20), the cortical density at the radius was significantly lower than in the healthy controls [19]. Our results are in line with these previous results in patients with subclinical PAD, as cortical parameters assessed at the distal radius (i.e. Ct.BMD and Ct.Th) were associated with both microvascular (Cutolo score) and macrovascular (DBI values) parameters.

The main figure of microvascular disease in scleroderma is digital ulcers; the various processes including fibrotic, vascular and inflammatory events in this disease may explain both the early damage affecting the microvessels and the macrovascular obliterative disease [34] and could also impact on bone [15]. The alteration of the microvessels through its clinical expression that is ischemic digital ulcers has been reported to be associated with acroosteolysis [35]. This relationship was further confirmed in another study showing the link between acroosteolysis and late nailfold videocapillaroscopic patterns [36]. In our study, microvessel damages reflected by Cutolo score ≥2 were included in the multivariate models being associated with both cortical density and thickness at the radius.

Although the vasculopathy in SSc has been described rather by an obliterative vasculopathy than a classical atherosclerosis [37]. Hypertension, diabetes, tobacco use, and hyperlipidaemia could have been confounding factors in our study. However, these cardiovascular risk factors known to exert also a possible role in bone metabolism were not significantly associated to the vascular parameters investigated in the present study. Vitamin D is also a potential confounder of the relationship between bone and vessels [38]. Vitamin D may likely intervene to simultaneously inhibit vascular calcification and bone loss [39]. In our study, we observed a vitamin D deficiency in 6% of patients and a vitamin D insufficiency in 54.5% of patients. However vitamin D levels were neither associated with bone parameters in univariate nor in multivariate analyses.

Developing arterial calcifications due to the deposition of hydroxyapatite in vascular tissues and simultaneously exhibit a bone loss has been called the “calcification paradox” [40]. Although it appears to be feasible to assess lower leg calcification during an HRpQCT exam [41], we have not investigated this parameter in our study. The mechanisms which are implicated in the occurrence of subcutaneous calcinosis are not clearly elucidated. In addition, their preferential localisation at the fingers in some occasion in parallel to acro osteolysis is also paradoxical. However, it was beyond the scope of our study to evaluate the site-matched analysis of subcutaneous calcification and acro-osteolysis in relation with microarchitecture at the radius. Nonetheless, the percentage of both acro osteolysis and subcutaneous calcinosis did not differed according to the DBI value (data not shown).

There is probably not a unifying mechanism which explains the complete relationship between PAD and osteoporosis. A decreased bone perfusion is probably one of the possible mechanism inducing bone loss through its ischemia-related phenomenon such as regional hypoxia, acidosis, and disturbance of vascular permeability which may in turn impact the hematopoietic stem cells quiescence [42]. Laroche *et al.* have reported in men with asymmetric PAD a decrease in bone mineral content in the more affected leg as compared to the other one [43]. Conversely to this observation, in a longitudinal study including elderly women, there was a relationship between BMD not only at the hip and calcaneus but also at the radius and ankle-brachial index [18] suggesting that a single localized phenomenon of reduced blood flow cannot explain the relationship between bone loss and vascular damages. Our study has some limitations such as the relatively small size of our population and the lack of control group. The cross-sectional nature of our study cannot authorize any causal relationship between the damage of micro and macrovasculature in SSc and bone microarchitectural alterations observed at the radius. Although the radius is a relevant site of study for osteoporosis purpose, its type of blood supply differs from that of vertebrae, and thus the bone damage observed in our study might be site specific and not a systemic one. Commonly used drugs in SSc such as statines, proton pump inhibitors and glucocorticoids were not considered as exclusion criteria, but herein the clinical size of their pharmacological effects is probably not significant. Patients in this study were all Caucasian women and thus results cannot be extrapolated to other population or ethnicity.

This study has also important strengths. First, this is the first study investigating the bone vascular axis in SSc using HRpQCT device. HRpQCT provides an in depth analysis of bone microarchitecture and volumetric density at both cortical and trabecular bone. Secondly, although the number of patients in the study was limited (*n* = 33), the studied population was relatively homogeneous regarding to the subtype of disease (26/33 had a limited cutaneous form). Third, our patients fulfilled both the ACR 1980 and the ACR/EULAR 2013 criteria.

In conclusion, our study findings, which show an association between the severity of microvessels and macrovessels damage and altered microarchitectural parameters at the radius in SSc, confirm the link between vessels and bone that has been evidenced in previous studies.

Longitudinal studies in larger group of patients with SSc might contribute to allow future preventive and therapeutic advice against osteoporosis in SSc patients with microvessels or macrovessels alterations.

## MATERIALS AND METHODS

### Study population

We conducted a single-center cross-sectional study including consecutive female patients with SSc, from April 2012 to February 2013, in the Rheumatology and Dermatology departments of the Hospital Regional Center of Orléans (France). The study was approved by the local Ethical Committee of Tours (No. 2012-R1) on March 27, 2012. Written informed consent was obtained from all study participants. The diagnosis of SSc was based on the criteria of the American College of Rheumatology (ACR)/European League Against Rheumatism (EULAR) 2013 and/or on the criteria of LeRoy and Medsger 2001 [44, 45]. The criteria of Leroy and Medsger allowed to differentiate limited and diffuse cutaneous form of SSc. Exclusion criteria were age younger than 18, pregnant or breastfeeding women. Table 5 provides an overview of the protocol visits and procedures.

**Table 5:** Schedule of activities

Study activities	Screening visit	Baseline visit
Informed consent	Χ	
Inclusion/exclusion criteria	Χ	Χ
General, cardiovascular, osteoporosis Specific medical history and prior/current medication use	Χ	
Primary diagnosis Demographics	Χ	
General physical examination	Χ	Χ
Laboratory		Χ
Nailfold videocapillaroscopy		Χ
Laser-Doppler ultrasonography		Χ
High resolution peripheral QCT		Χ

### Clinical and biological evaluation

Disease characteristics were reported: disease duration, disease subtype (limited or diffuse cutaneous SSc, or sine scleroderma SSc), overall functional disability measured with the Health Assessment Questionnaire (HAQ) and treatments such as DMARDs, antimalarials, Iloprost and Cyclophosphamide. Skin involvement was assessed using the modified Rodnan skin thickness score (mRSS) [46]. Joint involvement was defined by history or current arthralgia or arthritis. Interstitial lung disease is usually defined by the combination of at least 10–15% extent of lung fibrosis on HRCT scan and FVC <70–75%. In this paper, lung disease was defined by the presence of abnormalities on functional exploration, such as diffusing capacity for carbon monoxide lower than 75% or a total lung capacity lower than 80% and/or the presence of interstitial lung disease seen on chest X-Ray and/or chest Computed Tomography. Cardiac involvement was defined by a suspicion of pulmonary hypertension, characterized by a systolic pulmonary artery pressure ≥30 mmHg on transthoracic echography. Gastrointestinal involvement was defined by the presence of dysphagia, gastroesophageal reflux, malabsorption syndrome and abnormal oesophageal manometry. Renal involvement was assessed by serum creatinine and history of scleroderma renal crisis. Blood test was realized in all patients, including: blood count, serum electrolytes, serum creatinine and blood urea, C-reactive protein (CRP) and erythrocyte sedimentation rate (ESR), liver function tests, lipid profile, serum albumin, parathyroid hormone, 25-hydroxy-vitamin D. The vitamin D deficiency was defined as a serum 25-hydroxyvitamin D below 10 ng/ml, and vitamin D insufficiency by a rate between 10 and 30 ng/ml. The presence of specific antibodies in SSc was collected retrospectively: antinuclear factors, anticentromere, antitopoisomerase (antiScl70) and antiRNA polymerase III antibodies.

### Cardiovascular and osteoporosis risk factors

The following variables were collected: age, weight, height, diabetes, high blood pressure and/or dyslipidemia, smoking and alcohol consumption, menopause duration, personal history of low-energy fracture, family history of fracture of the femur upper extremity, current intake of proton pump inhibitors, current corticosteroid use or a weaning for less than one year and dose, calcium and/or vitamin D supplementation, anti-osteoporotic treatment and its duration.

### Evaluation of vascular assessment

Macroangiopathy of the upper limbs was investigated by laser-Doppler ultrasonography, performed at the day of patient inclusion by an experienced practitioner. The laser-Doppler measured the systolic pressure of the second and fourth fingers at the non-dominant hand (mmHg), with a wavelength of 780 nanometers including a pressure module *(PeriFlux 5000, PERIMED, Stockholm, Sweden),* associated to a pneumatic cuff with manometer for measuring brachial blood pressure. The measure of brachial blood pressure (mmHg) was used to calculate the DBI of the second and the fourth fingers, defined as the ratio of digital blood pressure to brachial blood pressure. A DBI less than 0.7 characterized the PAD. The permeability of radial and ulnar arteries and the functionality of palmar arches were also analysed.

Nailfold videocapillaroscopy was performed by an experienced practitioner, in each patient at the day of inclusion, using an optical probe with a ×100 magnification contact lens and connected to image analysis software (*CapXview HD, Xport technologies, Craponne, France).* The images were classified by Cutolo score [47] as normal (0) when there are regular distribution of capillaries without capillary loss, morphology without specific or aspecific changes, as early (I) when there are few giant capillaries, few capillary microhaemorrhages, no evident loss of capillaries, and relatively well-preserved capillary distribution, as active (II) when there are frequent giant capillaries, frequent capillary microhaemorrhages, moderate loss of capillaries, absent or mild ramified capillaries with mild disorganization of the capillary architecture, and as late (III) when there are few or absent giant capillaries and microhaemorrhages, severe loss of capillaries with extensive avascular areas, and neoangiogenesis defined by irregular enlargement of the capillaries, disorganization of the normal capillary array and ramified/bushy capillaries. For statistical analysis, patients were grouped according to their capillaroscopic patterns as non-severe (0-I) or severe involvement (II-III).

PAD was studied using vascular criteria extracted from the SSc severity score of Medsger 2003 [48]. This score defined the vascular involvement as follow: normal pattern as no Raynaud syndrome or Raynaud syndrome not requiring vasodilators (0), mild pattern as Raynaud syndrome requiring vasodilatators (1), moderate pattern as digital pitting scars (2), severe pattern as digital tip ulcerations (3), and endstage as digital gangrene (4). The PAD was considered as non-severe if the score was 0 to 1, and severe if the score was 2 to 4, as described in a previous study [49].

### HRpQCT imaging and analysis

vBMD and bone microarchitecture were measured by HRpQCT (*Xtrem CT, Scanco Medical AG, Brüttisellen, Switzerland*) at the non-dominant side of the distal radius. The forearm was immobilized in a splint made of carbon fiber to reduce motion artifacts. An anteroposterior scout view was used to define the measured area. The scan volume spanned 9.02 mm in length and started 9.5 mm from the reference line in the proximal direction as previously described [15]. All scans were reconstructed using an isotropic voxel sizes of 82 μm. The outcome variables used in our analysis were the following: total volumetric BMD (Tt.BMD) in mgHA/cm^3^, cortical volumetric BMD (Ct.BMD) in mgHA/cm^3^, trabecular volumetric BMD (Tb.BMD) in mgHA/cm^3^, trabecular thickness (Tb.Th) in μm, trabecular number (Tb.N) in mm-1, trabecular bone separation (Tb.Sp) in μm, cortical thickness (Ct.Th) in μm, intra-individual distribution of separation (Tb.Sp.SD) in μm, trabecular bone volume (BV/TV) in % [49].

### Statistical analysis

Analyses were realized using the R software Version 3.1.2 and R Studio Version 0.98.1091 – © 2009–2014 RStudio, Inc. The tests were performed in bilateral formulation. *p* values < 0.05 were considered to be significant. For the univariate analysis of the relationship between bone involvement and vascular parameters, binary variables were analyzed by a comparison of means by the Student *t* test for normally distributed variables, and by the Wilcoxon test in the other cases. A univariate analysis of the association between cardiovascular risk factors and vascular parameters was realized by Fisher test. Variables with *p* < 0.2 were included in the multivariate analysis [50]. Multivariate analysis was performed by multiple linear regression. The selection of variables was based on the minimization of the Bayesian Information Criterion (BIC) by a bidirectional step-by-step method. The adjusted coefficient R^2^ was the main benchmark for choosing the best model. Normality of residuals was verified by a graphical method (Quantile-Quantile graph) and controlled by the Shapiro and Wilk test. The quality of the models was assessed by monitoring the equivariance and independence of the residuals, and the research for influential or aberrant observations.
